# Targeting IRAK4 with Emavusertib in Lymphoma Models with Secondary Resistance to PI3K and BTK Inhibitors

**DOI:** 10.3390/jcm12020399

**Published:** 2023-01-04

**Authors:** Francesca Guidetti, Alberto J. Arribas, Giulio Sartori, Filippo Spriano, Laura Barnabei, Chiara Tarantelli, Reinhard Von Roemeling, Elizabeth Martinez, Emanuele Zucca, Francesco Bertoni

**Affiliations:** 1 Institute of Oncology Research, Faculty of Biomedical Sciences, USI, 6500 Bellinzona, Switzerland; 2 SIB Swiss Institute of Bioinformatics, 1015 Lausanne, Switzerland; 3 Curis, Inc., Lexington, MA 02421, USA; 4 Oncology Institute of Southern Switzerland, 6500 Bellinzona, Switzerland

**Keywords:** IRAK4, PI3K, BTK, marginal zone lymphoma, MYD88

## Abstract

Inhibitors of phosphatidylinositol 3-kinase (PI3K) and Bruton tyrosine kinase (BTK) represent a recognized option for the treatment of patients affected by indolent B cell lymphomas. However, small molecules as single agents show limited success in their ability in inducing complete responses, with only partial remission achieved in most patients, suggesting the need for combination therapies. IRAK4 is a protein kinase downstream of the Toll-like receptor signaling (TLR), a driver pathway of secondary tumor° resistance in both hematological and solid tumor malignancies. Activation of IRAK4 upon TLRs and IL-1 receptor (IL-1R) stimulation and through the adaptor protein MYD88 initiates a signaling cascade that induces cytokine and survival factor expression mediated by the transcription factor NF-κB. MYD88-L265P encoding mutations occur in diffuse large B-cell lymphomas, in lymphoplasmacytic lymphomas and in few marginal zone lymphomas (MZL). The IRAK4 inhibitor emavusertib (CA-4948) has shown early safety and clinical activity in lymphoma and leukemia patients. In this preclinical study, we assessed emavusertib effectiveness in MZL, both as single agent and in combination with targeted agents, with a particular focus on its capability to overcome resistance to BTK and PI3K inhibitors. We showed that the presence of MYD88 L265P mutation in bona fide MZL cell lines confers sensitivity to the IRAK4 inhibitor emavusertib as single agent. Emavusertib-based combinations improved the sensitivity of MZL cells to BTK and PI3K inhibitors, including cells with a secondary resistance to these agents. Emavusertib exerted its activity via inhibition of NF-κB signaling and induction of apoptosis. Considering the early safety data from clinical trials, our study identifies the IRAK4 inhibitor emavusertib as a novel compound to be explored in trials for patients with MYD88-mutated indolent B cell lymphomas as single agent and as combination partner with BTK or PI3K inhibitors in unselected populations of patients.

## 1. Introduction

The introduction of inhibitors of phosphatidylinositol 3-kinase (PI3K) and Bruton tyrosine kinase (BTK) has represented a big step forward in the treatment of patients affected by indolent B cell lymphomas, including marginal zone lymphoma (MZL) and lymphoplasmacytic lymphoma (LPL) [1,2,3,4,5,6,7]. Idelalisib was the first-in-class PI3Kδ inhibitor to be approved by the U.S. Food and Drug Administration (FDA), in combination with the anti-CD20 monoclonal antibody rituximab, for the treatment of indolent B cell lymphoma [4]. Additional PI3K-inhibitors such as copanlisib, a pan-class I PI3K inhibitor with predominant activity against PI3Kα/PI3Kδ, and umbralisib, the first-in-class dual phosphatidylinositol 3-kinase delta (PI3Kδ) and casein kinase 1 epsilon (CK1ε) inhibitor, also showed clinical activity in patients affected by indolent lymphomas [1,5,6]. Bruton’s Tyrosine Kinase (BTK) inhibitors, such as ibrutinib, are also effective in treating MZL and LPL patients [2,3,7]. However, these small molecules as single agents have shown limited success in their ability in inducing complete responses, with only partial remission achieved in most patients, suggesting the need for combination therapies [8].

IRAK4 is a protein kinase downstream the Toll-like receptor signaling (TLR), a known driver of secondary tumor resistance in both hematological and solid tumor malignancies [9]. Activation of IRAK4 upon TLRs and IL-1 receptor (IL-1R) stimulation and through the adaptor protein MYD88 initiates a signaling cascade that induces cytokine and survival factor expression mediated by the transcription factor NF-κB. Mutations of proteins within the TLR/IL-1R pathway can lead to an increased NF-κB activity and the promotion of cancer. For example, MYD88-L265P encoding mutations are known to occur in a fraction of the activated B cell (ABC) diffuse large B-cell lymphomas (DLBCL), in the vast majority of LPL, and in rare MZL, with differences based on the primary anatomical site [2,10,11,12,13,14,15,16,17]. In ABC DLBCL, MYD88-L265P leads to oncogenic activation of NF-κB through TLR-MYD88-IRAK1/4-TRAF6, independent of B cell receptor (BCR)–BTK activity and establishes a pathway for potential ibrutinib resistance [10]. Furthermore, in a large cohort of splenic MZL patients, mutations of NF-κB pathway in combination with an “immune-suppressive” phenotype define a cluster of patients with inferior relative survival [17].

To date, efforts to directly target MYD88 have largely been unsuccessful, and efforts have been so far focused on targeting IRAK4 [18,19,20,21,22,23]. Emavusertib (CA-4948) is a small molecule able to inhibit IRAK4 and to block TLR signaling. Emavusertib has shown single agent antitumor activity in ABC DLBCL, mantle cell lymphoma (MCL), and acute myeloid leukemia (AML) models [23,24,25,26,27], and in combination with ibrutinib (in ABC DLBCL, MCL), venetoclax and azacytidine (in AML) [24,25,26,27,28]. Early safety and clinical activity have been reported in both lymphoma and AML patients [29,30] and phase 1/2 trials are active for patients with hematologic cancers (NCT05178342, NCT04278768, NCT03328078).

In this preclinical study, we assessed emavusertib in MZL, both as single agent and in combination with targeted agents, with a particular focus on its capability to overcome resistance to BTK and PI3K inhibitors.

## 2. Methods

### 2.1. Cell Lines

Cell lines were cultured according to the recommended conditions, as previously described [31,32]. Cell line identity was periodically authenticated by short tandem repeat (STR) DNA profiling [33]. Cells were periodically tested to confirm Mycoplasma negativity using the MycoAlert Mycoplasma Detection Kit (Lonza, Visp, Switzerland).

### 2.2. Treatments

Response to single or drug combination treatments was assessed upon 72 h of exposure to increasing doses of drug followed by MTT assay, as previously described [34,35]. Idelalisib, copanlisib, umbralisib, ibrutinib were purchased from Selleckchem (Houston, TX, USA). Emavusertib was kindly provided by Curis (Lexington, MA, USA). To measure emavusertib effectiveness, IC50 was calculated using the four-parameter logistic regression. The beneficial effect of the combinations compared to the single agents was considered both as synergism according to the Chou-Talalay combination index [36] and as potency and efficacy according to the MuSyC algorithm [37]. The expected additivity was estimated based on the Bliss statement for drugs’ independence [38]. The parameter called “Benefit” was calculated as the difference between cell viability of the best single agent and the expected additivity (Bliss). The “Synergy” parameter was then calculated as the difference between the expected additivity values (Bliss) and the cell viability of each combination. Each parameter was calculated per each single combination of the two drugs. A given combination was considered “beneficial” with values of Benefit > 0; combinations with Benefit > 0 and Synergy ≈ 0 were considered “additive”; and combinations with Benefit > 0 and Synergy > 0 as “synergistic”. Antagonistic effect of a combination was given to values of Benefit < 0.

### 2.3. Flow Cytometry

Cell cycle was evaluated as previously reported [39]. Apoptosis assay was performed after 72 h treatment of emavusertib alone or in combination with ibrutinib by using eBioscience Annexin V Apoptosis Detection Kit (Thermo Fisher Scientific, Basel, Switzerland). Apoptosis induction and cell cycle distribution after the treatment were evaluated with flowCore R package from Bioconductor.

### 2.4. Immunofluorescence and Confocal Microscopy

Immunofluorescence and confocal microscopy were conducted as already reported [40]; cells were treated for 6 h and coated on a poly-L-lysine matrix then fixed 20 min with Paraformaldehyde (PFA) 4% at room temperature (RT). Cells were permeabilized with PBS + 0.1% Triton X-100 10 min at RT. To block unspecific staining, samples were blocked for 1 h with 5% BSA (TBST) at RT before staining. Antibodies were diluted in 5% Bovine Serum Albumin (BSA) (TBST). Samples were incubated overnight at 4 °C with primary antibody rabbit monoclonal anti human NF-κB p65 (D14E12) (1/100; Cell Signaling). Secondary goat antibody anti-rabbit IgG labelled with Alexa 568 (Thermo Fisher Scientific) 1 h at RT in the dark. Slides were counterstained after 3 washes of PBS with 0.3 μg/mL 4,6-diamidino-2-phenylindole (Sigma-Aldrich, Buchs, Switzerland). Images were acquired on a Leica SP5 with an objective with ×63 magnification. Protein quantification and NF-κB1/p65 were evaluated by ImageJ software.

## 3. Results

### 3.1. The IRAK4 Inhibitor Emavusertib Is Beneficial in MYD88 Mutated Lymphoma Cells

**Figure 1:**
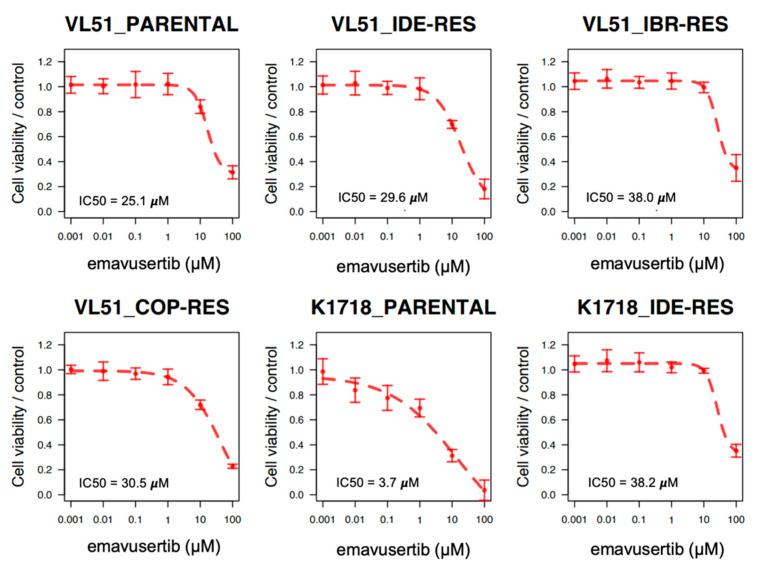
IRAK4 inhibitor emavusertib as single agent in MZL models. Dose–response curves (from three independent experiments), error bars and IC50 values of emavusertib in Karpas1718 (K1718) and VL51 parental cell lines or resistant models to idelalisib (IDE-RES) ibrutinib (IBR-RES) or copanlisib (COP-RES). Cells were exposed (72 h) to increasing doses of emavusertib as single agent followed by MTT assay. IC50 values were calculated using the four-parameter logistic regression.

The IRAK4 inhibitor emavusertib was tested in two marginal zone lymphoma models (VL51 and Karpas1718) [41,42] and their derivatives with secondary resistance to PI3K and BTK inhibitors [32,43,44,45]. Emavusertib determined a dose-dependent reduction in cell proliferation in all the cells. The most sensitive cell line was the parental Karpas1718 cell line, a bona fide MZL cell line bearing the MYD88 L265P mutation, with an IC50 of 3.72 µM. Conversely, IC50 values were only in the range of 21–38 µM in the VL51 cell line and its three derivatives, as well as in the Karpas1718 derivative (Figure 1). Overall, emavusertib did not show a significant increased activity in MZL cell lines resistant compared to parental condition (Figure 1).

### 3.2. Emavusertib Increases Sensitivity to PI3K and BTK Inhibitors in Resistant Models of MZL

**Figure 2:**
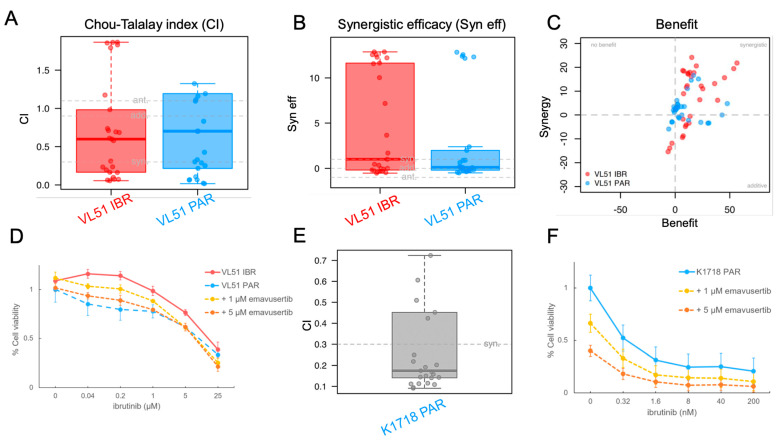
IRAK4 inhibitor emavusertib in Karpas1718 parental (K1718) and VL51 parental (PAR) and ibrutinib-resistant line (IBR). Cells were exposed (72 h) to increasing doses of ibrutinib alone or in combination with emavusertib followed by MTT assay. (**A**) Chou-Talalay index (CI). Each dot represents the CI value for a single ratio of ibrutinib/emavusertib. syn, synergism (CI < 0.9); add, additive effect [0.9 < CI < 1.1]; ant, antagonism/no benefit (CI > 1.1). (**B**) Synergistic efficacy (syn eff) calculated according to MuSyC algorithm. Each dot represents the syn eff value for a single ratio of ibrutinib/emavusertib. syn, synergism (syn eff > 1); add, additive effect [-1 < syn eff < 1]; ant, antagonism/no benefit (syn eff < -1). (**C**) Summary plot of benefit in terms of additivity or synergy as described in the method section. (**D**) Drug-response curves in VL51 ibrutinib-resistant cells upon emavusertib combination with ibrutinib. (**E**) Chou-Talalay index (CI) and (**F**) Drug-response curves in K1718 parental line treated (72 h) with ibrutinib alone or in combination with increasing doses of emavusertib.

**Figure 3:**
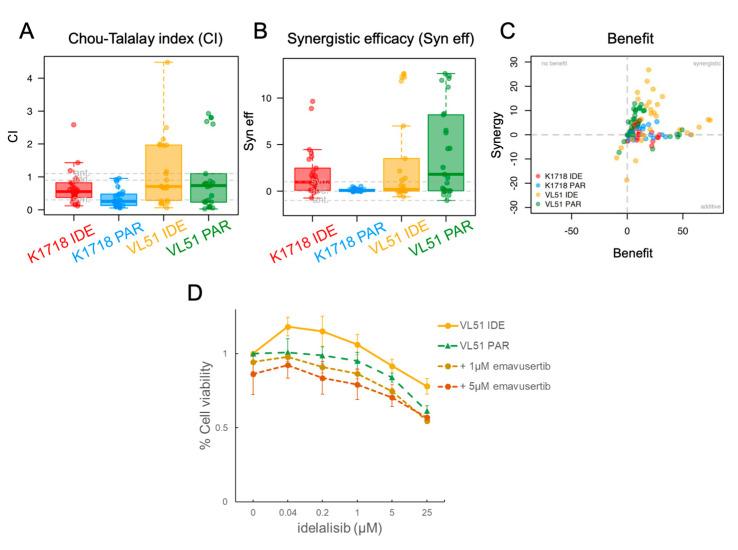
IRAK4 inhibitor emavusertib in VL51 and Karpas1718 (K1718) parental (PAR) and idelalisib-resistant (IDE) lines. Cells were exposed (72 h) to increasing doses of idelalisib alone or in combination with emavusertib followed by MTT assay. (**A**) Chou-Talalay index (CI). Each dot represents the CI value for a single ratio of idelalisib/emavusertib. syn, synergism (CI < 0.9); add, additive effect [0.9 < CI < 1.1]; ant, antagonism/no benefit (CI > 1.1). (**B**) Synergistic efficacy (syn eff) calculated according to MuSyC algorithm. Each dot represents the syn eff value for a single ratio of idelalisib/emavusertib. syn, synergism (syn eff > 1); add, additive effect [−1 < syn eff < 1]; ant, antagonism/no benefit (syn eff < −1). (**C**) Summary plot of benefit in terms of additivity or synergy as described in the method section. (**D**) Drug-response curves in VL51 idelalisib-resistant and parental lines upon emavusertib and idelalisib combination.

**Figure 4:**
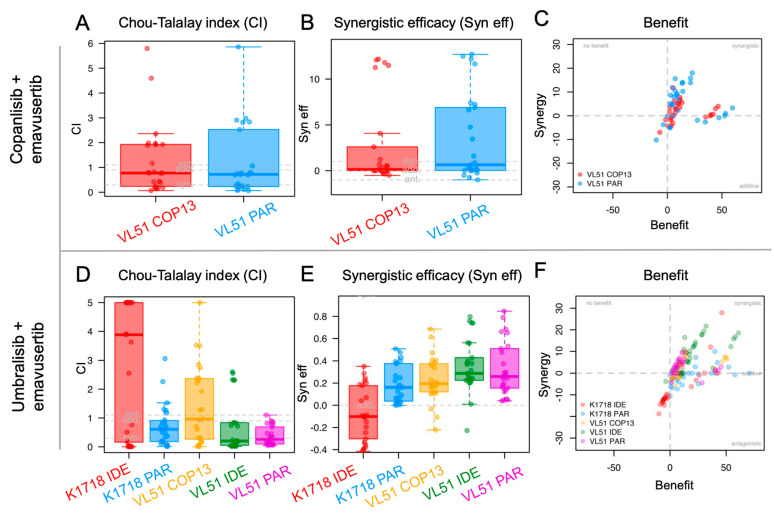
IRAK4 inhibitor emavusertib in combination with copanlisib and umbralisib in MZL cell lines. Upper panel: combination of emavusertib and copanlisib in VL51 parental (PAR) and copanlisib-resistant (COP13) line. Lower panel: combination of emavusertib and umbralisib in VL51 and K1718 parental (PAR) and resistant models to copanlisib (COP13), idelalisib (IDE) or ibrutinib (IBR). In both panels, cells were exposed (72 h) to increasing doses of PI3K inhibitors alone and in combination with emavusertib followed by MTT. (**A**,**D**): syn, synergism (CI < 0.9); add, additive effect [0.9 < CI < 1.1]; ant, antagonism/no benefit (CI > 1.1). (**B**,**E**): syn, synergism (syn eff > 1); add, additive effect [−1 < syn eff < 1]; ant, antagonism/no benefit (syn eff < −1). Each dot represents the CI value for a single ratio of copanlisib/emavusertib (upper panel) or umbralisib/emavusertib (lower panel). (**C**,**F**): Summary plot of benefit in terms of additivity or synergy, as described in the method section, for the combinations of emavusertib with copanlisib (upper panel) or umbralisib (lower panel).

**Figure 5:**
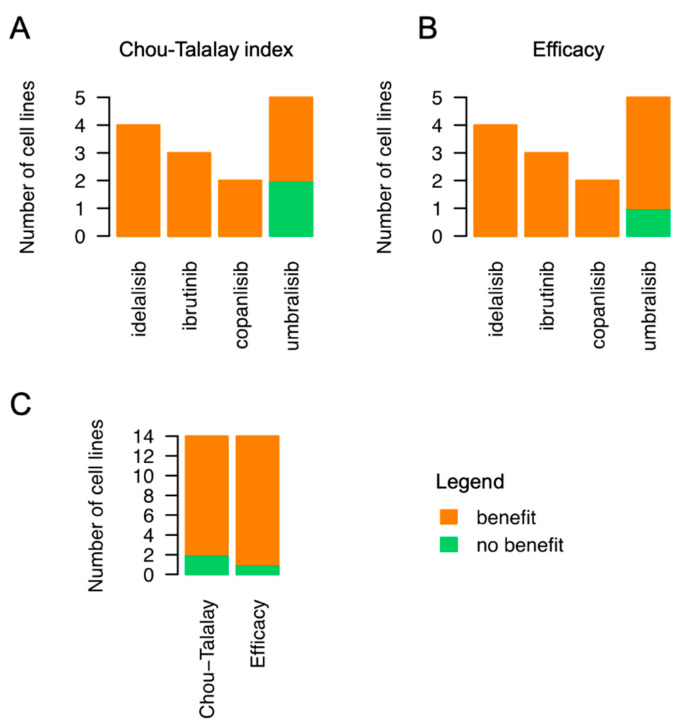
Combination of IRAK4 inhibitor emavusertib and BCR-signaling targeting agents is beneficial (synergistic or additive) in 12/14 models tested. Summary plots of all combinations tested in MZL parental and resistant lines. Cells were exposed (72 h) to increasing doses of idelalisib, ibrutinib, copanlisib or umbralisib alone or in combination with increasing doses of emavusertib followed by MTT assay. Synergy scores from Chou-Talalay (**A**) and MuSyc (Efficacy, **B**) models identified beneficial effects on the addition of emavusertib to inhibitors of downstream BCR signaling. (**C**) Summary of synergy models for all combinations.

We then assessed emavusertib in combination with BTK and PI3K inhibitors in the parental cells and in their derivatives with secondary resistance. Emavusertib was strongly synergistic with ibrutinib especially in the VL51 ibrutinib resistant model compared to the parental one (Figure 2A–C). The addition of emavusertib (from 1 to 5 µM) restored the sensitivity to ibrutinib in the ibrutinib-resistant cells reaching IC50 values comparable to the parental counterpart (Figure 2D). A strong anti-proliferative activity of the emavusertib-ibrutinib combination was also observed in the ibrutinib-sensitive Karpas1718 parental line (Figure 2E–F). Similarly, emavusertib was also synergistic in combination with idelalisib (Figure 3A–C) and restored sensitivity to idelalisib in idelalisib-resistant VL51 cells (Figure 3D). Comparably to what was observed with the ibrutinib combination, emavusertib doses from 1 to 5 µM were able to revert resistance and increase sensitivity to idelalisib in the idelalisib-resistant VL51 model (Figure 3D). The combination with idelalisib in Karpas1718 model, although beneficial, revealed no major advantages in the resistant model compared to the already sensitive parental counterpart (Figure 3A–C). The addition of emavusertib to copanlisib was more beneficial in the VL51 parental compared to resistant copanlisib-resistant derivative, but the increase in the efficacy was limited (Figure 4A–C). The combination of emavusertib with umbralisib was synergistic in VL51 parental and idelalisib-resistant, and in the parental Karpas1718, while exhibited no benefit in copanlisib-resistant VL51 and in idelalisib-resistant Karpas1718 (Figure 4D–F). Synergy, according to the Chou-Talalay index (CI < 0.9), and an increased synergistic efficacy, based on the MuSyC algorithm (syn eff > 1), were achieved in 86% (*n* = 12/14) and 93% (*n* = 13/14), respectively, of the MZL models tested (Figure 5). In summary, the addition of the IRAK4 inhibitor emavusertib was beneficial to the anti-tumor activity of PI3K and BTK inhibitors and it can also overcome the acquired resistance in MZL models.

### 3.3. Emavusertib Affects Proliferation and Induces Apoptosis in Both Sensitive and Resistant Marginal Zone Lymphoma Models

**Figure 6:**
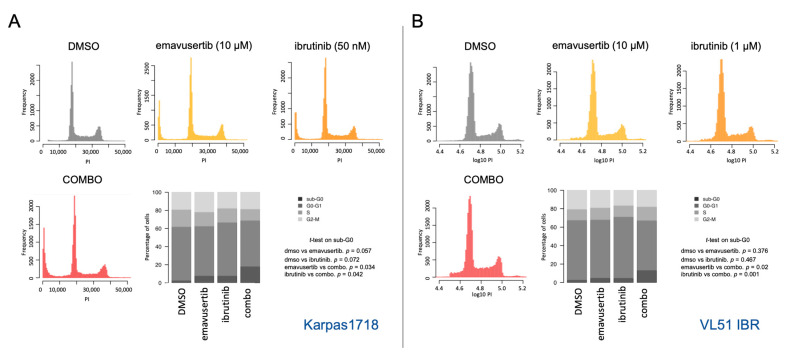
Emavusertib in combination with ibrutinib increases sub G0-G1 population. Representative histograms and cell-cycle distribution upon emavusertib (10 µM) alone or in combination with ibrutinib (72 h) in (**A**) K1718 parental and (**B**) VL51 ibrutinib resistant cell line. Ibrutinib doses were selected based on the IC50 values in the parental lines (50 nM in K1718, 1 µM in VL51). Data represent the average of two independent experiments. *p* for nominal *p*-value from t-test.

**Figure 7:**
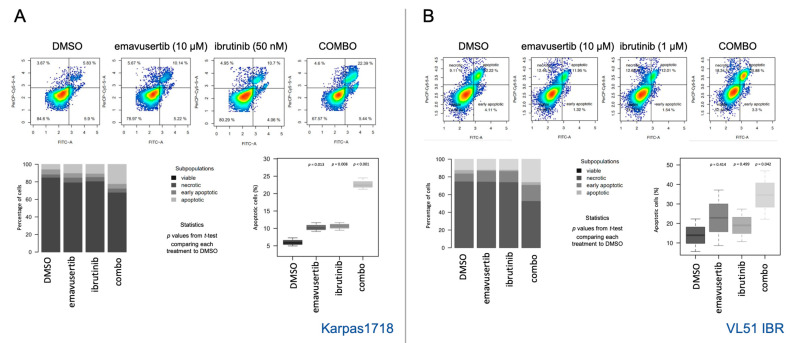
Addition of emavusertib to ibrutinib induces apoptosis. Apoptosis induction after emavusertib (10 µM) alone or in combination with ibrutinib (72 h) in (**A**) K1718 and (**B**) VL51 ibrutinib resistant line. Ibrutinib doses were selected based on the IC50 values in the parental lines (50 nM in K1718, 1 µM in VL51). Data represent the average of two independent experiments. Error bars for standard deviation of the mean. *p* for nominal *p*-value from t-test comparing each treatment to control (DMSO).

Based on drug-response results of the combinations, we further investigated the beneficial effect of combining emavusertib with the BTK inhibitor ibrutinib, being the most promising combination. We focused on the VL51 ibrutinib-resistant model, which demonstrated the higher advantage from the combination compared to the parental cell line, and on Karpas1718 parental cell line which was the most sensitive model to both emavusertib and ibrutinib. We further determined whether the pharmacological inhibition of IRAK4 affected cell cycle distribution using flow cytometry. Emavusertib treatment (10 μM) for 72 h decreased the percentage of proliferating cells and induced a moderate increase in the sub-G0 fraction, especially in the combination compared to single treatments (*p* < 0.05) (Figure 6A,B). Accumulation of cells in sub-G0 indicates DNA fragmentation and cell death possibly driven by apoptotic processes. Indeed, emavusertib (10 μM, 72 h) induced significant increase in apoptotic cell population, particularly when combined to ibrutinib compared to the single agents (Figure 7A,B). Decreased viability upon treatment was paired with a significant increase in apoptotic cells (*p* < 0.05) in both parental Karpas1718 (Figure 7A) and ibrutinib resistant VL51 (Figure 7B).

### 3.4. Emavusertib Reduces Total and Nuclear REL-A in MZL Cells

**Figure 8:**
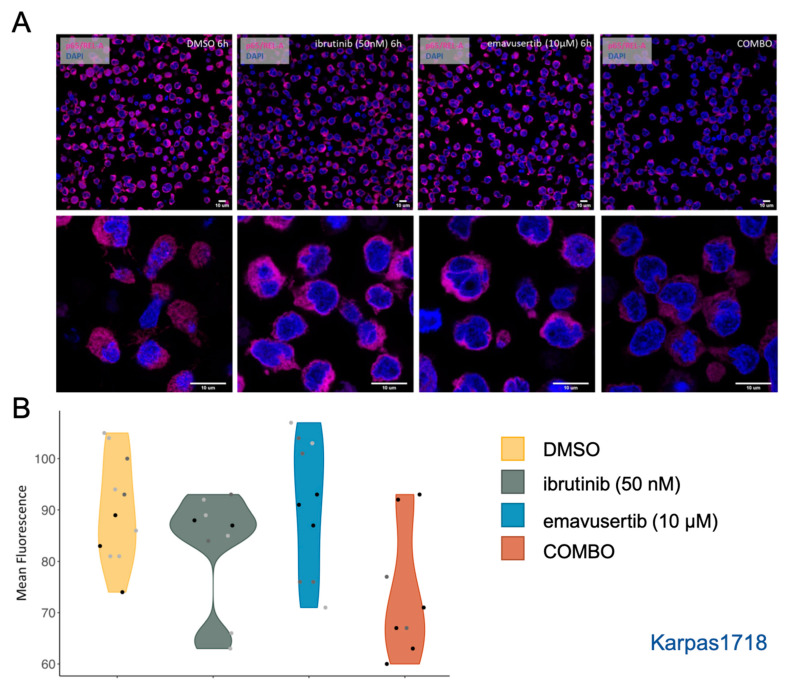
Combination of emavusertib with ibrutinib reduces p65/REL-A. (**A**) Representative immunofluorescence pictures and (**B**) Violin plot showing mean fluorescence quantification by confocal microscopy using specific anti-RELA/p65 (Magenta) and DAPI (4′-6-diamidino-2-phenylindole). Images were acquired on a Leica SP5 with an objective with ×63 magnification. Emavusertib (10 µM) was used alone or in combination with ibrutinib (50 nM) (6 h) in K1718 parental model. Data represent the mean fluorescence values. Scales of grey represent different experiments.

Both emavusertib and ibrutinib have already demonstrated the capability to modulate and decrease the activation of NF-κB signaling pathway which is known to be responsible for proliferation and tumorigenesis [23]. We investigated the mechanism underlying the observed synergy focusing on NF-κB signaling cascade by emavusertib (10 µM) and/or ibrutinib (50 nM) exposure in the parental Karpas1718. By immunofluorescence, we observed a consistent reduction in total p65/REL-A in the combination already after 6 h of ibrutinib and emavusertib combination (Figure 8A,B).

## 4. Discussion

Here, we showed that the presence of MYD88 L265P mutation, in bona fide MZL cell lines, confers sensitivity to the IRAK4 inhibitor emavusertib as single agent, emavusertib-based combinations can improve the sensitivity of MZL cells to BTK and PI3K inhibitors, also after acquisition of a secondary resistance to these agents, and emavusertib exerts its activity via inhibition of NF-κB signaling and induction of apoptosis. 

Driven by the central role of NF-κB signaling and MYD88 in MZL, we studied the pharmacological inhibition of IRAK4 with emavusertib in two MZL cell lines and their derivatives with acquired resistance to FDA approved PI3K and BTK inhibitors. Emavusertib showed the strongest dose-dependent anti-proliferative activity in the Karpas1718 cell line bearing mutated MYD88. Albeit a fraction of MZL patients present MYD88 abnormalities [2,10,11,12,13,14], the prevalence of these mutations, in particular the L265P, is much higher in LPL, [2,10,11,12,13,14,15,16,17] and we cannot rule out the possibility that the cell line derives from a LPL rather than from MZL. Nonetheless, our results are in line with the previously reported activity of emavusertib in MYD88 L265P mutated ABC DLBCL cell lines [26], and they indicate that emavusertib as single agent could be explored in the population of patients with MYD88 mutated LPL or MZL.

The activity of the IRAK4 inhibitor decreased in the Karpas1718 derivative cells which, after long exposure to the PI3Kδ inhibitor idelalisib, activated an ERBB4-mediated signaling leading to acquired resistance to multiple BTK and PI3K inhibitors [43]. No relevant differences were observed in the VL51 MZL cell line between parental and its three derivatives obtained by long exposure to idelalisib, ibrutinib or copanlisib and driven by overexpression of IL6, IL16 and IL1, respectively [32,44,45].

When given in combination with BTK (ibrutinib) and PI3K inhibitors (idelalisib, copanlisib and umbralisib), emavusertib increased the sensitivity of MZL cells to the targeted agents both in the parental and resistant clones. The beneficial effect of adding the IRAK4 inhibitor was particularly striking in the ibrutinib-resistant model we studied (VL51). In this cell line, nor emavusertib nor ibrutinib alone were able to induce an anti-proliferative response while the combination of the two compounds proved to be beneficial. Our data extend the reported preliminary data combining emavusertib with ibrutinib in ABC-DLBCL and MCL and with venetoclax and azacytidine in AML [24,25,26,27,28] that led to the early combination clinical trials [29,30].

As a whole, the addition of the IRAK4 inhibitor appeared to improve the efficacy (i.e., maximal effect) rather than the potency (i.e., minimal active dose) of the combination partners. The fact that benefit was achieved without increase in the potency may have positive implication in enhancing the therapeutic efficacy keeping the off-target effects low [37].

Considering the early safety data from clinical trials [29,30], our study identifies the IRAK4 inhibitor emavusertib as a novel compound to be explored as single agent in trials for patients with MYD88-mutated indolent B cell lymphomas and as combination partner with BTK or PI3K inhibitors in unselected populations of patients.

## References

[1] Dreyling M., Santoro A., Santoro A., Mollica L., Leppa S., Follows G., Lenz G., Kim W.S., Nagler A., Dimou M. (2020). Long-term safety and efficacy of the PI3K inhibitor copanlisib in patients with relapsed or refractory indolent lymphoma: 2-year follow-up of the CHRONOS-1 study. Am. J. Hematol..

[2] Noy A., de Vos S., Coleman M., Martin P., Flowers C.R., Thieblemont C., Morschhauser F., Collins G.P., Ma S., Peles S. (2020). Durable ibrutinib responses in relapsed/refractory marginal zone lymphoma: Long-term follow-up and biomarker analysis. Blood Adv..

[3] Tam C.S., Opat S., D’Sa S., Jurczak W., Lee H.P., Cull G., Owen R.G., Marlton P., Wahlin B.E., Sanz R.G. (2020). A randomized phase 3 trial of zanubrutinib vs ibrutinib in symptomatic Waldenström macroglobulinemia: The ASPEN study. Blood.

[4] Wagner-Johnston N.D., Schuster S.J., de Vos S., Salles G., Jurczak W.J., Flowers C.R., Viardot A., Flinn I.W., Martin P., Xing G. (2021). Outcomes of patients with up to 6 years of follow-up from a phase 2 study of idelalisib for relapsed indolent lymphomas. Leuk. Lymphoma.

[5] Panayiotidis P., Follows G.A., Mollica L., Nagler A., Özcan M., Santoro A., Stevens D., Trevarthen D., Hiemeyer F., Garcia-Vargas J. (2021). Efficacy and safety of copanlisib in patients with relapsed or refractory marginal zone lymphoma. Blood Adv..

[6] Davids M.S., O’Connor O.A., Jurczak W., Samaniego F., Fenske T.S., Zinzani P.L., Patel M.R., Ghosh N., Cheson B.D., Derenzini E. (2021). Integrated safety analysis of umbralisib, a dual PI3Kδ/CK1ε inhibitor, in relapsed/refractory lymphoid malignancies. Blood Adv..

[7] Trotman J., Buske C., Tedeschi A., Matous J.V., MacDonald D., Tam C.S., Tournilhac O., Ma S., Treon S.P., Oriol A. (2021). Single-Agent Ibrutinib for Rituximab-Refractory Waldenström Macroglobulinemia: Final Analysis of the Substudy of the Phase III Innovate(TM) Trial. Clin. Cancer Res..

[8] Bertoni F., Rossi D., Raderer M., Zucca E. (2020). Marginal Zone Lymphomas. Cancer J..

[9] Rhyasen G.W., Starczynowski D.T. (2015). IRAK signalling in cancer. Br. J. Cancer.

[10] Ngo V.N., Young R.M., Schmitz R., Jhavar S., Xiao W., Lim K.H., Kohlhammer H., Xu W., Yang Y., Zhao H. (2011). Oncogenically active MYD88 mutations in human lymphoma. Nature.

[11] Li Z.M., Rinaldi A., Cavalli A., Mensah A.A., Ponzoni M., Gascoyne R.D., Bhagat G., Zucca E., Bertoni F. (2012). MYD88 somatic mutations in MALT lymphomas. Br. J. Haematol..

[12] Cascione L., Rinaldi A., Bruscaggin A., Tarantelli C., Arribas A.J., Kwee I., Pecciarini L., Mensah A.A., Spina V., Chung E.Y.L. (2019). Novel insights into the genetics and epigenetics of MALT lymphoma unveiled by next generation sequencing analyses. Haematologica.

[13] Yan Q., Huang Y., Watkins A.J., Kocialkowski S., Zeng N., Hamoudi R.A., Isaacson P.G., de Leval L., Wotherspoon A., Du M.Q. (2012). BCR and TLR signaling pathways are recurrently targeted by genetic changes in splenic marginal zone lymphomas. Haematologica.

[14] Wu F., Watanabe N., Tzioni M.M., Akarca A., Zhang C., Li Y., Chen Z., Cucco F., Carmell N., Noh J.Y. (2021). Thyroid MALT lymphoma: Self-harm to gain potential T-cell help. Leukemia.

[15] Alaggio R., Amador C., Anagnostopoulos I., Attygalle A.D., Araujo I.B.O., Berti E., Bhagat G., Borges A.M., Boyer D., Calaminici M. (2022). The 5th edition of the World Health Organization Classification of Haematolymphoid Tumours: Lymphoid Neoplasms. Leukemia.

[16] de Leval L., Alizadeh A.A., Bergsagel P.L., Campo E., Davies A., Dogan A., Fitzgibbon J., Horwitz S.M., Melnick A.M., Morice W.G. (2022). Genomic profiling for clinical decision making in lymphoid neoplasms. Blood.

[17] Bonfiglio F., Bruscaggin A., Guidetti F., Terzi di Bergamo L., Faderl M., Spina V., Condoluci A., Bonomini L., Forestieri G., Koch R. (2022). Genetic and phenotypic attributes of splenic marginal zone lymphoma. Blood.

[18] Saikh K.U. (2021). MyD88 and beyond: A perspective on MyD88-targeted therapeutic approach for modulation of host immunity. Immunol. Res..

[19] Erika Shiratori M.I., Ohtaka M., Nogami S., Tohda S. (2016). Mechanisms of Suppressive Effects of MYD88 Inhibitors on the Growth of Lymphoma and Leukemia Cells. Blood.

[20] Zhang J., Fu L., Shen B., Liu Y., Wang W., Cai X., Kong L., Yan Y., Meng R., Zhang Z. (2020). Assessing IRAK4 Functions in ABC DLBCL by IRAK4 Kinase Inhibition and Protein Degradation. Cell Chem. Biol..

[21] Chen Y., Bai G., Ning Y., Cai S., Zhang T., Song P., Zhou J., Duan W., Ding J., Xie H. (2020). Design and synthesis of Imidazo[1,2-b]pyridazine IRAK4 inhibitors for the treatment of mutant MYD88 L265P diffuse large B-cell lymphoma. Eur. J. Med. Chem..

[22] Kelly P.N., Romero D.L., Yang Y., Shaffer A.L., Chaudhary D., Robinson S., Miao W., Rui L., Westlin W.F., Kapeller R. (2015). Selective interleukin-1 receptor-associated kinase 4 inhibitors for the treatment of autoimmune disorders and lymphoid malignancy. J. Exp. Med..

[23] Gummadi V.R., Boruah A., Ainan B.R., Vare B.R., Manda S., Gondle H.P., Kumar S.N., Mukherjee S., Gore S.T., Krishnamurthy N.R. (2020). Discovery of CA-4948, an Orally Bioavailable IRAK4 Inhibitor for Treatment of Hematologic Malignancies. ACS Med. Chem. Lett..

[24] Booher R.N., Samson M.E.S., Borek M., Modafferi H., Martell R.E. (2019). PS991. CA-4948, an IRAK4/FLT3 inhibitor, shows antileukemic activity in mouse models of *FLT3* wild-type and *FLT3* mutated Acute Myeloid Leukemia (AML). Hemasphere.

[25] von Roemeling C.A., Doonan B.P., Hoang-Minh L., Tun H.W., Martinez E., Soikes R., von Roemeling R., Mitchell D.A. (2021). Abstract P243: The IRAK4 inhibitor CA-4948 demonstrates antitumor activity in a preclinical model of CNS lymphoma. Mol. Cancer Ther..

[26] Booher R.N., Samson M.E., Xu G.-X., Cheng H., Tuck D.P. (2017). Abstract 1168: Efficacy of the IRAK4 inhibitor CA-4948 in patient-derived xenograft models of diffuse large B cell lymphoma. Cancer Res..

[27] Booher R.N., Nowakowski G.S., Patel K., Lunning M.A., Samson M.E.S., Atoyan R., Ma A.W., Xu G.X., Dellarocca S., Modafferi H. (2018). Preclinical Activity of IRAK4 Kinase Inhibitor CA-4948 Alone or in Combination with Targeted Therapies and Preliminary Phase 1 Clinical Results in Non-Hodgkin Lymphoma. Blood.

[28] Ugolkov A., Hok R., von Roemeling R., Martell R.E. (2021). EP390. IRAK4 inhibitor CA-4948 potentiates antitumor effects of azacitidine and venetoclax in human acute myeloid leukemia. HemaSphere.

[29] Joffe E., Nowakowski G.S., Tun H.W., Rosenthal A.C., Lunning M.A., Ramchandren R., Li C.-C., Zhou L., Martinez E., Roemeling R.W.V. (2022). Open-label, dose-escalation, and expansion trial of CA-4948 in combination with ibrutinib in patients with relapsed or refractory hematologic malignancies. J. Clin. Oncol..

[30] Garcia-Manero G., Winer E.S., DeAngelo D.J., Tarantolo S.R., Sallman D.A., Dugan J., Groepper S., Giagounidis A., Gotze K.S., Metzeler K. (2022). Phase 1/2a study of the IRAK4 inhibitor CA-4948 as monotherapy or in combination with azacitidine or venetoclax in patients with relapsed/refractory (R/R) acute myeloid leukemia or lyelodysplastic syndrome. J. Clin. Oncol..

[31] Spriano F., Chung E.Y.L., Gaudio E., Tarantelli C., Cascione L., Napoli S., Jessen K., Carrassa L., Priebe V., Sartori G. (2019). The ETS Inhibitors YK-4-279 and TK-216 Are Novel Antilymphoma Agents. Clin. Cancer Res..

[32] Arribas A.J., Napoli S., Cascione L., Sartori G., Barnabei L., Gaudio E., Tarantelli C., Mensah A.A., Spriano F., Zucchetto A. (2022). Resistance to PI3Kdelta inhibitors in marginal zone lymphoma can be reverted by targeting the IL-6/PDGFRA axis. Haematologica.

[33] Gaudio E., Tarantelli C., Spriano F., Guidetti F., Sartori G., Bordone R., Arribas A.J., Cascione L., Bigioni M., Merlino G. (2020). Targeting CD205 with the antibody drug conjugate MEN1309/OBT076 is an active new therapeutic strategy in lymphoma models. Haematologica.

[34] Tarantelli C., Gaudio E., Arribas A.J., Kwee I., Hillmann P., Rinaldi A., Cascione L., Spriano F., Bernasconi E., Guidetti F. (2018). PQR309 Is a Novel Dual PI3K/mTOR Inhibitor with Preclinical Antitumor Activity in Lymphomas as a Single Agent and in Combination Therapy. Clin. Cancer Res..

[35] Boi M., Gaudio E., Bonetti P., Kwee I., Bernasconi E., Tarantelli C., Rinaldi A., Testoni M., Cascione L., Ponzoni M. (2015). The BET Bromodomain Inhibitor OTX015 Affects Pathogenetic Pathways in Preclinical B-cell Tumor Models and Synergizes with Targeted Drugs. Clin. Cancer Res..

[36] Chou T.C. (2010). Drug combination studies and their synergy quantification using the Chou-Talalay method. Cancer Res..

[37] Meyer C.T., Wooten D.J., Paudel B.B., Bauer J., Hardeman K.N., Westover D., Lovly C.M., Harris L.A., Tyson D.R., Quaranta V. (2019). Quantifying Drug Combination Synergy along Potency and Efficacy Axes. Cell Syst.

[38] Bliss C.I. (1939). The toxicity of poisons applied jointly. Ann. Appl. Biol..

[39] Mensah A.A., Kwee I., Gaudio E., Rinaldi A., Ponzoni M., Cascione L., Fossati G., Stathis A., Zucca E., Caprini G. (2015). Novel HDAC inhibitors exhibit pre-clinical efficacy in lymphoma models and point to the importance of CDKN1A expression levels in mediating their anti-tumor response. Oncotarget.

[40] Barnabei L., Lamrini H., Castela M., Jeremiah N., Stolzenberg M.-C., Chentout L., Jacques S., Bouafia A., Magérus-Chatinet A., Moncan M. (2020). Heterozygous RELA mutations cause early-onset systemic lupus erythematosus by hijacking the NF-κB pathway towards transcriptional activation of type-I Interferon genes. bioRxiv.

[41] Inokuchi K., Abo J., Takahashi H., Miyake K., Inokuchi S., Dan K., Nomura T. (1995). Establishment and characterization of a villous lymphoma cell line from splenic B-cell lymphoma. Leuk. Res..

[42] Martinez-Climent J.A., Sanchez-Izquierdo D., Sarsotti E., Blesa D., Benet I., Climent J., Vizcarra E., Marugan I., Terol M.J., Sole F. (2003). Genomic abnormalities acquired in the blastic transformation of splenic marginal zone B-cell lymphoma. Leuk. Lymphoma.

[43] Arribas A.J., Napoli S., Gaudio E., Cascione L., Di Veroli A., Tarantelli C., Spriano F., Zucchetto A., Rossi F.M., Rinaldi A. (2019). Secreted Factors Determine Resistance to Idelalisib in Marginal Zone Lymphoma Models of Resistance. Blood.

[44] Arribas A.J., Napoli S., Gaudio E., Cascione L., Veroli A.D., Tarantelli C., Spriano F., Zucchetto A., Rossi F., Sartori G. (2019). Abstract A127: Secretion of IL16 is associated with resistance to ibrutinib in pre-clinical models of lymphoma. Mol. Cancer Ther..

[45] Arribas A., Napoli S., Cascione L., Gaudio E., Bordone-Pittau R., Barreca M., Sartori G., Tarantelli C., Spriano F., Rinaldi A. (2020). Secondary resistance to the PI3K inhibitor copanlisib in marginal zone lymphoma. Eur. J. Cancer.

